# RETRACTION: Nestin Regulates Autophagy‐Dependent Ferroptosis Mediated Skeletal Muscle Atrophy by Ubiquitinating MAP 1LC3B

**DOI:** 10.1002/jcsm.70235

**Published:** 2026-02-08

**RETRACTION**: S. Han , X. Zhao , C. Yu , C. Cui , Y. Zhang , Q. Zhu , M. Qiu , C. Yang , and H. Yin , “Nestin Regulates Autophagy‐Dependent Ferroptosis Mediated Skeletal Muscle Atrophy by Ubiquitinating MAP 1LC3B,” Journal of Cachexia, Sarcopenia and Muscle 16, no. 2 (2025): e13779, 10.1002/jcsm.13779.40183241 PMC11969254

The above article, published online on 4 April 2025 in Wiley Online Library (wileyonlinelibrary.com), has been retracted by agreement between the authors; the journal Editor‐in‐Chief, Stefan D. Anker; and Wiley Periodicals LLC. The retraction has been agreed upon following concerns raised by a third party. An investigation identified duplications between images included in Figure 5C of this article and images previously published in another article by some of the same authors. These duplicated images were used to represent a different scientific context. Further duplications were identified between elements of Figures 1D and S5A, Figures 8D and S6D and Figures S4F and S5A. The authors cooperated with the investigation and explained that the duplications were a result of figure mismanagement. They also provided some supporting data. However, the information provided was insufficient to restore the editors' confidence in the results and conclusions. Hence, the editors who handled the paper, together with the Editor‐in‐Chief, can no longer consider the results and conclusions of this article to be valid.

